# Supplementary figures and images for: TSSKL is essential for sperm mitochondrial morphogenesis and male fertility in moths
Source: PLoS Genet. 2025 Oct 24;21(10):e1011914. doi: 10.1371/journal.pgen.1011914 (PMC12551868; doi:10.1371/journal.pgen.1011914)

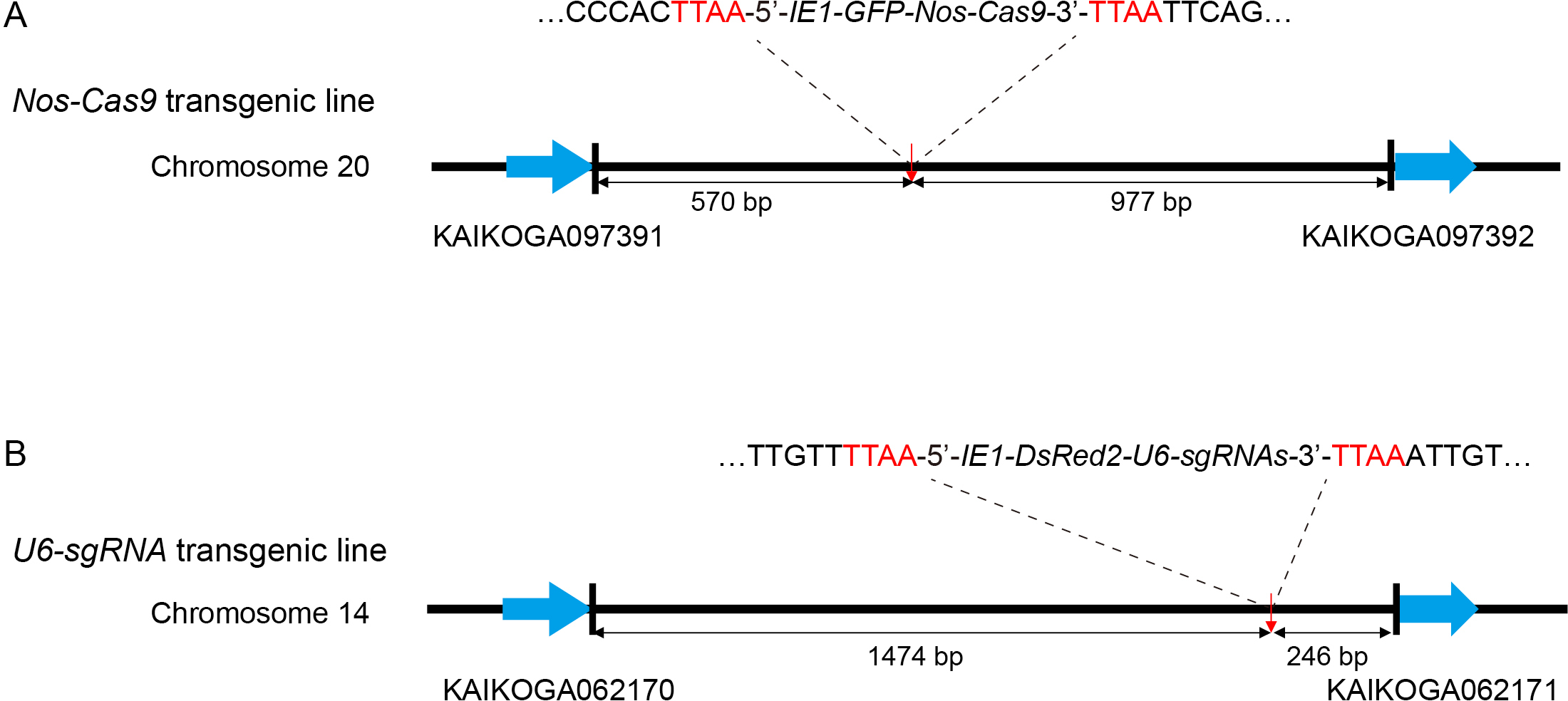

Supplement: S1 Fig — The insertion sites are marked by vertical red arrows. The flanking genes are annotated with blue arrows, and the distances from the insertion sites to these genes are labeled in base pairs. (TIF) [file pgen.1011914.s001.tif]

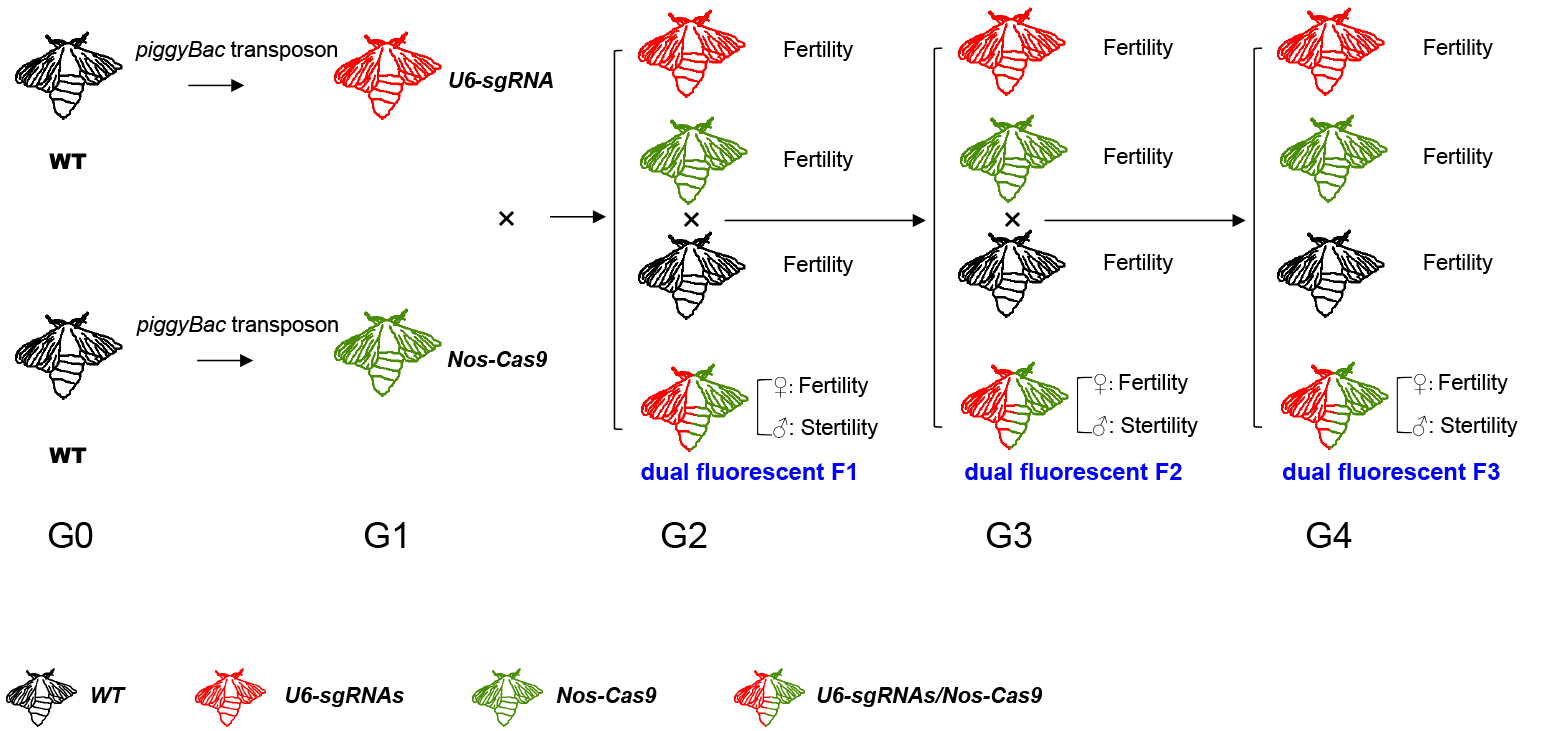

Supplement: S2 Fig — Schematic of crosses done to demonstrate heritably of the male sterility phenotype in B. mori. The activator line (Nos-Cas9) is represented as a green moth, the effector line (U6-sgRNAs) is in red, the positive line is in half green and half red, and the negative line is in black. Four lines are produced by the hybridization of the Nos-Cas9 line with the U6-sgRNAs line in B. mori. (TIF) [file pgen.1011914.s002.tif]

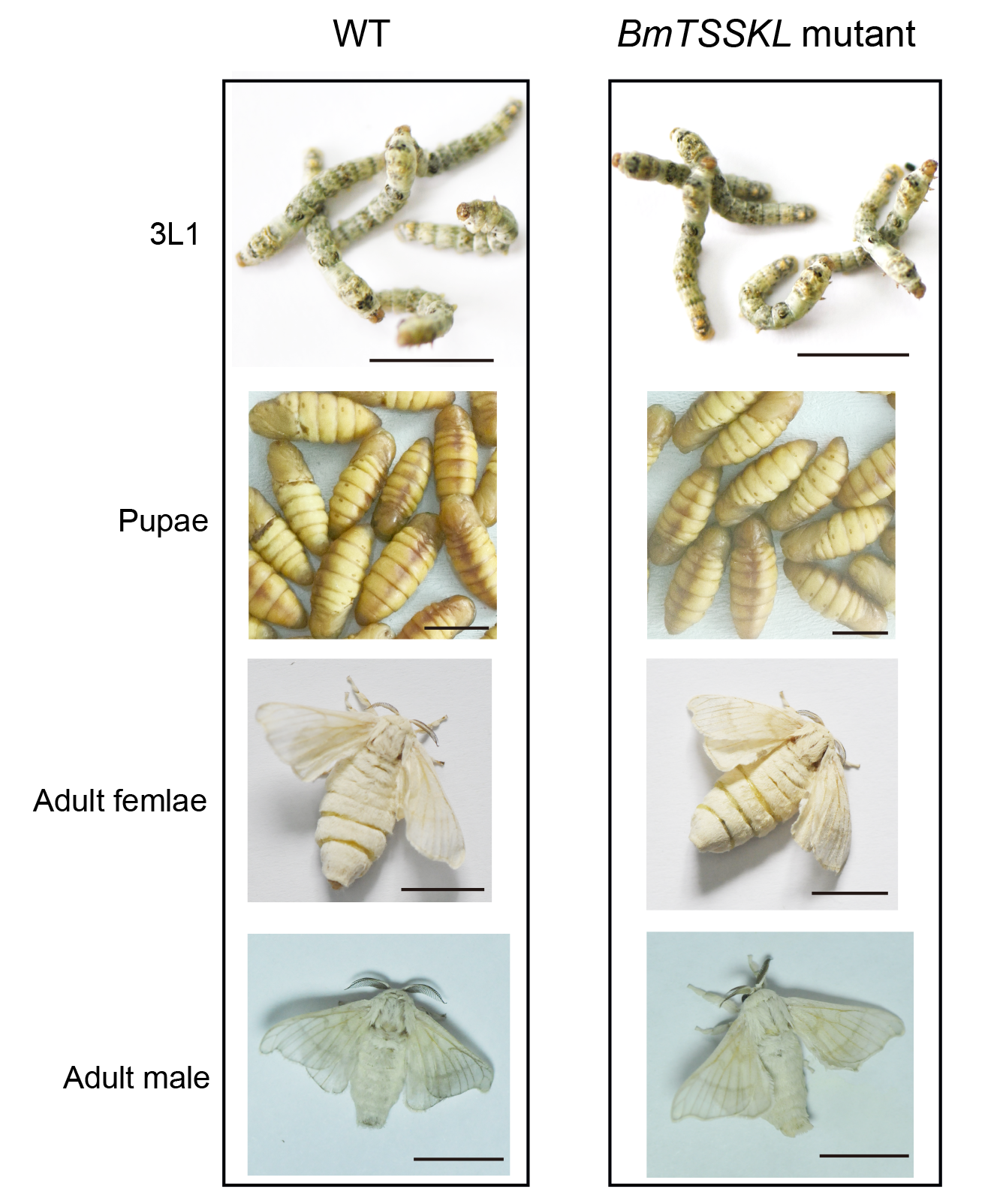

Supplement: S3 Fig — Scale bars: 1 cm. (TIF) [file pgen.1011914.s003.tif]

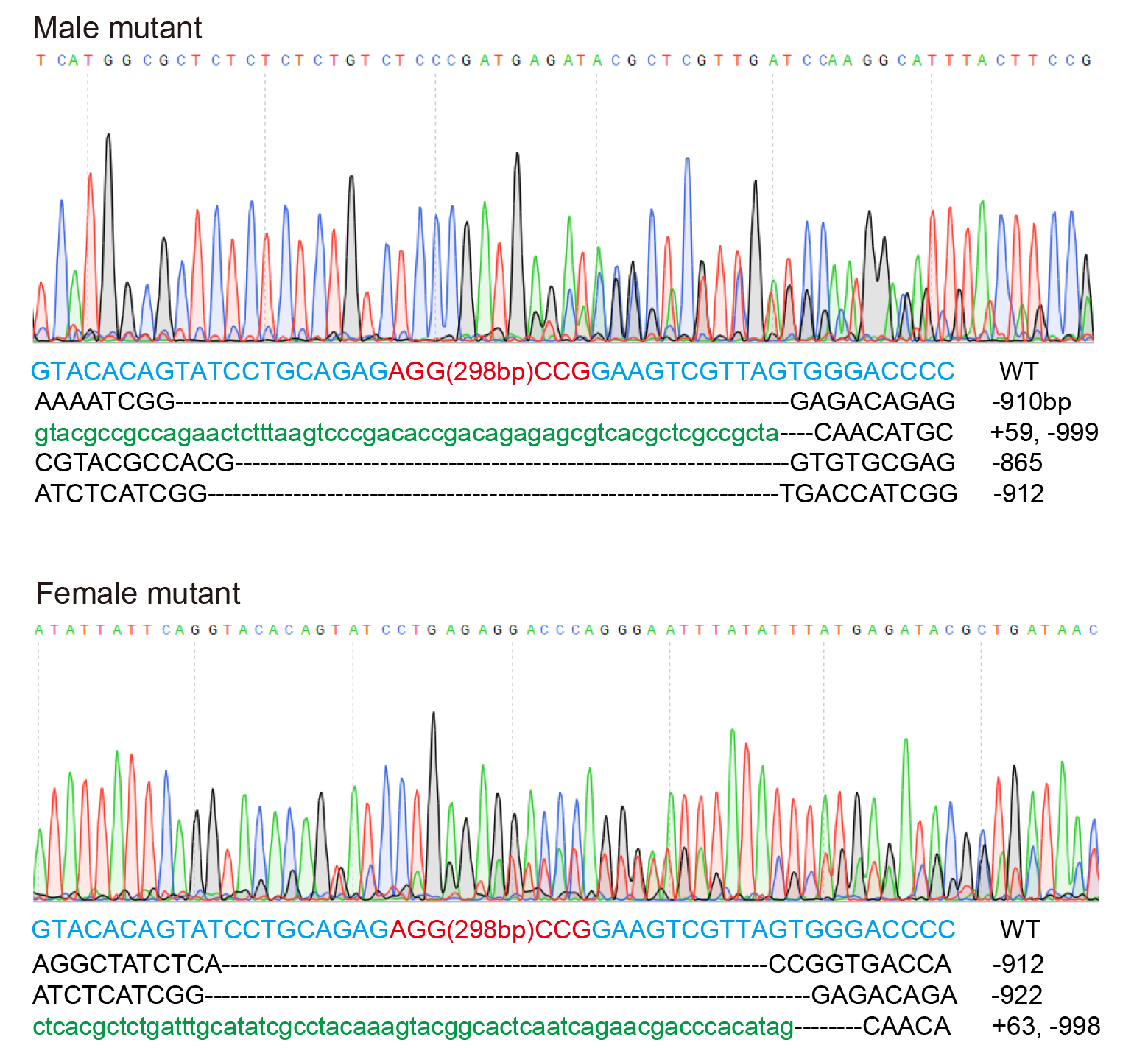

Supplement: S4 Fig — Various deletions or insertions mutations were detected in injected males and females. Dashed lines represent the deleted bases, the green lowercase letters represent the inserted bases, and PAM are highlighted in red. (TIF) [file pgen.1011914.s004.tif]

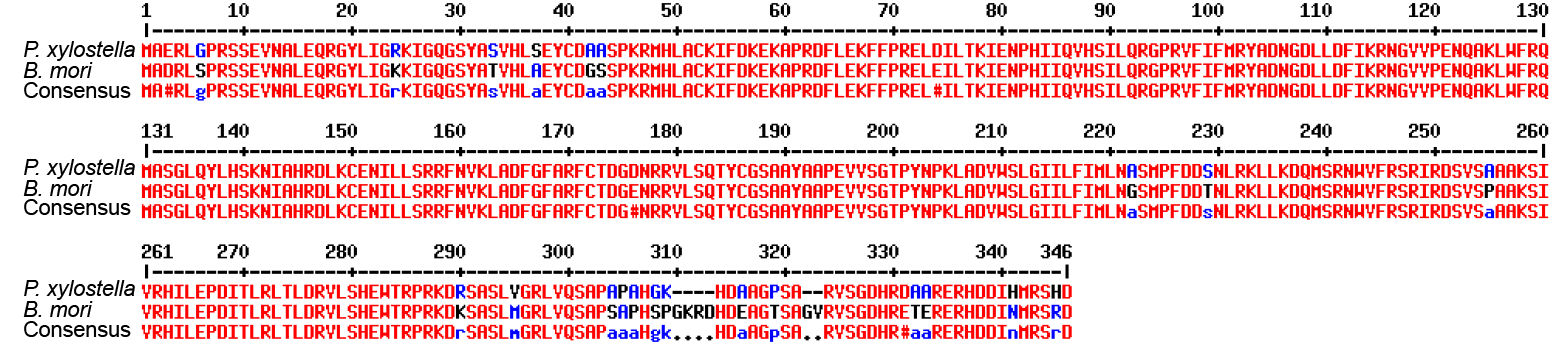

Supplement: S5 Fig — (TIF) [file pgen.1011914.s005.tif]

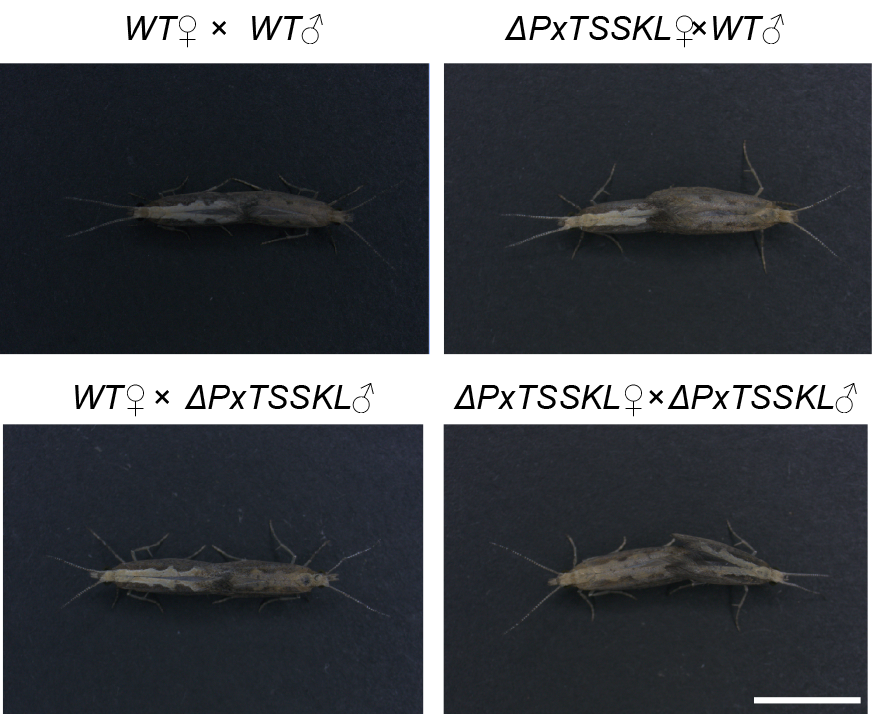

Supplement: S6 Fig — WT males mated with WT and PxTSSKL mutant females, PxTSSKL mutant males mated with WT and PxTSSKL mutant females. Scale bar, 5 mm. (TIF) [file pgen.1011914.s006.tif]
