# Supplementary material for: TSSKL is essential for sperm mitochondrial morphogenesis and male fertility in moths
Source: PLoS Genet. 2025 Oct 24;21(10):e1011914. doi: 10.1371/journal.pgen.1011914 (PMC12551868; doi:10.1371/journal.pgen.1011914)
Supplement: Attachment — Submitted filename: Response to Reviews.pdf [file pgen.1011914.s013.pdf]

Editorial level reviewer

1.“Data not shown” statements are present throughout the text and this is not permitted. The authors either need to show the data supporting these conclusions or remove these claims entirely.

Response: Thanks for your suggestions. We have added new results regarding the morphological differences between WT and *BmTSSKL* mutants at different developmental stages (larvae, pupae, adult females and males) in the revised manuscript. (See S2 Fig).

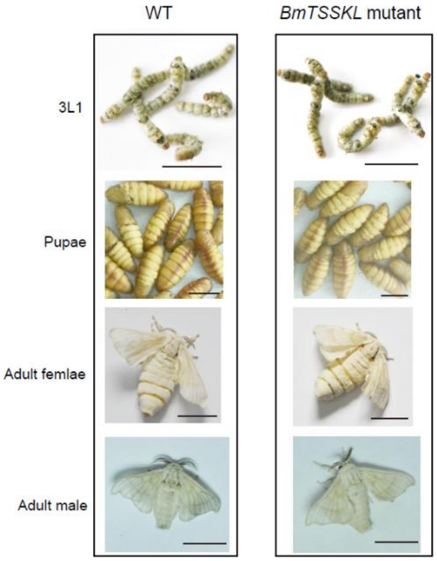

2. It is not entirely clear how the authors validated their mutants. They seem to be using a G0 CRISPR approach where the nos-cas9 x U6-sgRNA-TSSKL progeny are used and called “mutants” but it does not appear that a single mutant KO line was derived for the experiments. The authors need to explicitly show genotyping information for the mutants and confirm that the female germline is also edited.

Additionally, if G0s are being analyzed, the nos-cas9 would not drive KO anywhere but in the germ cells, so the authors cannot make conclusions about somatic roles of TSSKL. Several other related concerns were raised by the reviewers regarding the KOs. It is difficult to interpret the results without the nature of the mutations being made via CRISPR being defined and validated.

Response: We apologize for the issues of description and presentation in the original manuscript that might cause confusion for you and another reviewer. We have added the S1 Fig and S3 Fig to provide comprehensive details of our CRISPR/Cas9 mutagenesis strategy. To confirm the correlation between phenotypic defects and mutations in *BmTSSKL*, we amplified and sequenced the

BmTSSKL **gene from the testis of injected males with abnormal fertility and the ovary of injected females with normal fertility. Heterozygous chromatograms and various deletions or insertions were observed in the** ***BmTSSKL*** **mutant individuals of both sexes. Relevant representative results have been added in S3 Fig (Lines 164-168, and 497-500).**

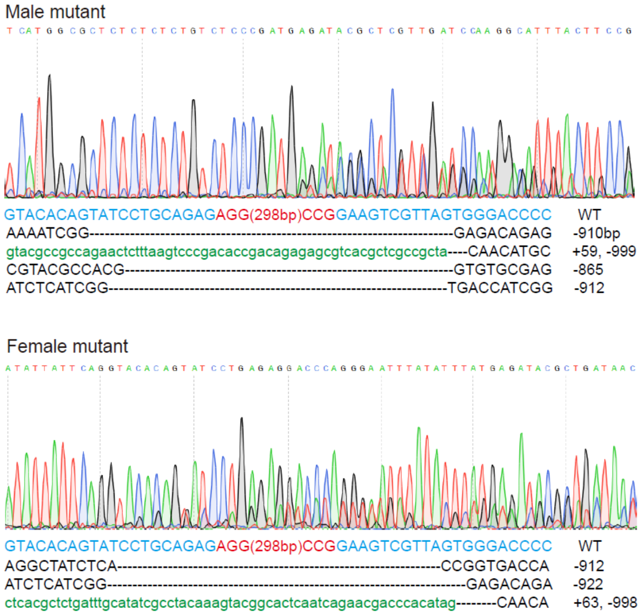

In addition, to enhance the scientific rigor of the manuscript, we have implemented the following revisions:

"Subsequently, the ***U6-sgRNA*** **(*TSSKL*) and** ***Nos-Cas9*** **lines were crossed to generate the** ***TSSKL*** **mutant line (Fig 1G and S1 Fig). Genomic DNA was then extracted from randomly selected dual fluorescent (dsRed and GFP) individuals, and the target regions were PCR-amplified and sequenced. Sequencing analysis revealed the occurrence of various large deletions in the** ***BmTSSKL*** **transgenic line (Fig 1H). These results demonstrated that we have successfully obtained the** ***BmTSSKL*** **deficiency mutants." (Lines 136-141).**

"To generate the TSSKL mutant, we carried out silkworm germline transformation by microinjecting Nos-Cas9 and U6-sgRNA respectively into the pre-blastoderm Nistari embryos along with a mixture of piggyBac helper vectors. Then, embryos (generation 0: G0) were incubated in a humidified chamber at 25 °C until hatching. The resulting larvae were reared to adulthood and backcrossed with WT moths to produce progeny. Through fluorescence microscopy screening, we selected G1

individuals displaying single fluorescent markers (GFP or DsRed) and intercrossed them to obtain G2 progeny. This crossing strategy yielded four distinct germlines: (1) dual fluorescent mutants (GFP/DsRed) expressing both Cas9 and sgRNA, (2) Nos-Cas9 line (GFP/+), (3) U6-sgRNA (DsRed/+), and (4) non-transgenic controls (fluorescence-negative) (S1 Fig). Meanwhile. the double-positive individuals (GFP/DsRed) were identified as putative mutants and selected for subsequent genotypic validation." (Lines 352-363 and 486-491).

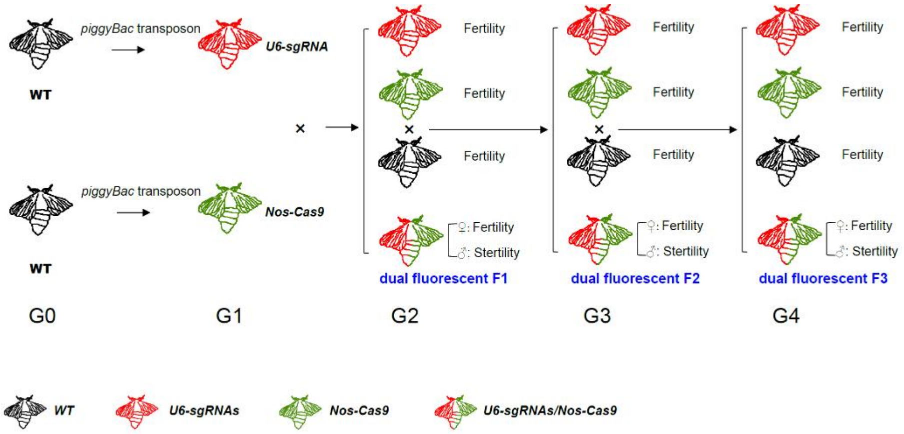

3. The number of eggs laid is not sufficient evidence to draw conclusions about female fertility in the mutants. The authors need to describe how they distinguish unhatched eggs from unfertilized eggs before inferring that the TSSKL KO impacts fertility.

Response: We appreciate your constructive comments. Our results demonstrated that both female and male *TSSKL* mutants exhibited normal mating behavior (Fig 2E-2H). Typically, a female silkworm moth lays approximately 350 ± 20 eggs after mating, with a hatching rate of ~95%. In our egg-laying assays, both mutant and wild-type moths successfully mated. If the *TSSKL* mutation had no effect on fertility, the expected hatching rate should have remained at 95%. However, experimental groups containing male *TSSKL* mutants, the egg hatching rate was 0% (Fig 2B), indicating that the *TSSKL* mutation cause male-specific sterility. Although we did not directly examine the fertilization status of these unhatched eggs, two potential explanations emerge: i) Insufficient sperm transfer to the spermatheca, leading to fertilization failure (Fig 3B), or ii) Sperm defects rendering them incapable of fertilization (Fig 3D and 3E). Notably, while we did not directly examine

the fertilization status of these eggs, this limitation does not affect our primary conclusion that *TSSKL* disruption leads to abnormal mitochondrial development and energy metabolism defects in sperm, ultimately leading to male sterility (Lines 145-148, 181-184 and 192-201). Indeed, this is an important point. It might be a good topic for our future research.

4. Experimental replicates and n values are not included anywhere in the manuscript, and statistics are not sufficiently defined/explained.

Response: Thank you for your advice. We have checked the entire manuscript and added the relevant descriptions in the revised manuscript.

5. The exact accession number / ID for the precise “BmTSSKL” in the study is not included anywhere.

Response: We appreciate your suggestion and have added accession number PX116274 in the revised manuscript (Line 120).

6. There are general concerns about clarity and organization of the manuscript, including inconsistencies between the text and figures, and the Discussion section needs major revisions.

Response: Thanks for your valuable comments. We have carefully refined the Discussion to improve its scientific rigor and comprehensiveness.

Reviewer #1

In this study, Xu et al. utilized CRISPR/Cas9-mediated mutagenesis to characterize the functional role of the *TSSKL* gene in the lepidopteran insects. *TSSKL* knockout induced complete male sterility and severe morphological defects in both eupyrene and apyrene sperm. Comparative transcriptome and functional analyses linked these phenotypes to dysregulated energy metabolism pathways. Their results provided novel insights into insect reproductive biology.

The manuscript is concise, but sufficiently clear written. Experiments seem to be properly designed and precisely conducted. Results obtained are significant and well support conclusions made. I have not found any serious shortcomings, I suggest only some minor revisions.

RESPONSE: We appreciate your high evaluation and thoughtful comments on our study. We have carefully addressed your concerns as below.

Minor suggestions

Lines 164-166: *BmTSSKL* mutant males mated with WT or *BmTSSKL* mutant females produced eggs normally but did not hatch, whereas WT males mated with WT or *BmTSSKL* mutant females laid eggs and hatched normally (Fig 2A). This sentence seems to have a grammatical error.

Response: Thanks for your comments. We have revised the description as follows:

"*BmTSSKL* mutant males mated with either WT or *BmTSSKL* mutant females produced normal egg yields, but these eggs failed to hatch (0%). Whereas WT males mated with either WT or *BmTSSKL* mutant females produced normal egg yields with successful hatching (94-96%)." (Lines 145-148).

Lines 212-214: we analyzed the mRNA expression of sperm motility genes at different developmental stages in WT and BmTSSKL mutant individuals was compared by using qRT-PCR. The meaning of this sentence seems unclear. Please check it.

Response: Thank you for your suggestion. To enhance clarity and eliminate potential ambiguity, we have revised the sentence as follows:

"we compared mRNA expression levels of sperm motility-related genes at different developmental stages between wild-type and *BmTSSKL* mutant individuals using qRT-PCR analysis." (Lines 185-188).

For *BmTSSKL*, which number is the accession ID of this gene in GenBank. Please add the number in the manuscript.

Response: We appreciate your suggestion and have added accession number PX116274 in the revised manuscript (Line 120).

In Supporting information, the file “S1 Table” was named as Table S2, “S2 Table” named as Table S3, and “S3 Table” named as Table S1. Please check them carefully.

Response: Thank you. We apologize for the mistake and have corrected it in the revised manuscript.

Reviewer #2

In this manuscript, the authors elucidate the role of TSSKL in sperm mitochondrial morphogenesis in lepidopterans. Their findings demonstrate that TSSKL deficiency leads to severe morphological abnormalities in both eupyrene sperm and apyrene sperm, associated with disrupted mitochondrial dynamics and aberrant autophagy activation. Furthermore, RNA-seq analysis revealed impaired mitochondrial metabolism in the testes of TSSKL mutant males. Overall, this study provides novel mechanistic insights into insect reproductive genetics. However, several aspects of the manuscript require improvement, as outlined below:

Response: We appreciate your positive comments on our study and further suggestions that help improve the manuscript.

1. The study reports consistent phenotypic effects in both *Bombyx mori* and *Plutella xylostella*. To strengthen the evolutionary context, please provide sequence alignment data.

Response: Thank you for your advice. We have conducted protein sequence alignment analyses, as presented in S4 Fig.

2. For *P. xylostella* egg counting: Given the species' reproductive biology, please detail your quantification methodology and any statistical validation of sampling approaches.

Response: Thank you for your kind reminding. We have added the relevant descriptions to the Methods section and corresponding figure legends. (Lines 402 and 597-599).

3. Regarding mutagenesis strategies: Explain the rationale for using double mutants in *B. mori* versus single mutants in *P. xylostella*, including any technical constraints.

Response: Thank you for your query. In this study, we successfully generated *TSSK* mutants in both *B. mori* and *P. xylostella* using CRISPR/Cas9-mediated gene editing. For *B. mori*, we employed the binary transgenic CRISPR/Cas9 system. In *P. xylostella*, however, technical constraints limited us to generating only sgRNA strains, as the large size of Cas9 mRNA (>4000 bp) presented significant challenges in obtaining stable Cas9 transgenic lines. While we were unable to establish a complete CRISPR/Cas9 transgenic system in *P. xylostella*, this limitation does not compromise the reliability or accuracy of our experimental findings.

4. Expand the discussion on how *TSSKL*-mediated mitochondrial dysfunction specifically impacts both sperm types.

Response: We sincerely appreciate your constructive suggestion. We have expanded the Discussion section with additional details. These modifications provide more comprehensive interpretation of our findings and strengthen the scientific rigor of our conclusions (Lines 298-304).

5. Discussion needs to be improved. Systematically integrate all examined genes into a proposed *TSSKL* regulatory network.

Response: We sincerely appreciate your constructive suggestions. We have enhanced the Discussion by adding detailed explanations (Lines 293-310).

6. Figure 1B, In the selected tissues, why are OV, TE, FO and MO? Can the influence of other gonads be excluded?

Response: We appreciate this insightful question regarding our tissue selection for expression analysis. OV (Ovary) and TE (testis) represented the primary reproductive tissues where TSSKL's function would be most critical. FO (whole body of 2 days old virgin adult females excluding the ovary) and MO (whole body of 2 days old virgin adult males excluding the testis) provided whole-adult expression excluding the OV or TE. We agree that examining additional reproductive tissues (e.g., spermathecae) could provide further insights and will consider this for future studies. The current data robustly demonstrate *TSSKL*'s reproductive-specific expression pattern while controlling for potential confounding factors.

7. Figure 2A and 5E, what is the explanation for the unhatched eggs in the mating between female mutants and wild-type individuals?

Response: We observed no significant difference in hatching success between WT and mutant females when mated with WT males ($p > 0.05$). In addition, *TSSKL* was significantly enriched in testes. These results conclusively demonstrate that *TSSKL* mutation does not impair female fertility, as the reproductive capacity of mutant females (egg production, fertilization competence, and embryonic support) remains statistically indistinguishable from WT controls.

8. Figure 2C, I suggest asterisks be added to denote significance.

Response: We have added the asterisks as suggested in Figure 2C (Line 547).

9. Figure 5G, the meaning of **** has not been explained.

Response: We have added the relevant information in the caption of Figure 5G (Line 597).

Reviewer #3

This study by Xu et al., identified TSSKL as an essential regulator in sperm mitochondrial morphogenesis and male fertility of moths. Their genetic and molecular experiments are well designed, and the data are presented clearly and concisely. Altogether, this is a nice study that uses two species to demonstrate the conserved role of TSSKL in sperm functions. However, I have some comments and concerns that should be addressed.

Response: We appreciate your overall positive comments on our study and further suggestions that help improve the manuscript. We have addressed all your comments as responded below.

1. Line 155: The authors state, "We confirmed that gene editing was successful by crossing Nos-Cas9 line with EGFP marker (Fig. 1G). However, it means that a Nos-Cas9/GFP line was used in combination with sgRNA/dsRed to induce TSSKL deletion or mutation. This strategy seems to serve as a method for generating or selecting TSSKL knockout/KO animals, rather than as a means of confirming successful gene editing.

Response: We are deeply grateful for your thoughtful suggestions. To enhance the scientific rigor of the manuscript, we have added the S1 Fig and implemented the following revisions:

"Subsequently, the sgRNA (*TSSKL*)-dsRed and Nos-Cas9 lines were crossed to generate the *TSSKL* mutant line (Fig 1G and S1 Fig). Genomic DNA was then extracted from randomly selected dual fluorescent (dsRed and GFP) individuals, and the target regions were PCR-amplified and sequenced. Sequencing analysis revealed the occurrence of various large deletions in the *BmTSSKL* transgenic line (Fig 1H). These results demonstrated that we have successfully obtained the *BmTSSKL* deficiency mutants." (Lines 136-141).

"To generate the TSSKL mutant, we carried out silkworm germline transformation by microinjecting *Nos-Cas9* and *U6-sgRNA* respectively into the pre-blastoderm Nistari embryos along with a mixture of piggyBac helper vectors. Then, embryos (generation 0: G0) were incubated in a humidified chamber at 25 °C until hatching. The resulting larvae were reared to adulthood and backcrossed with WT moths to produce progeny. Through fluorescence microscopy screening, we selected G1

individuals displaying single fluorescent markers (GFP or DsRed) and intercrossed them to obtain G2 progeny. This crossing strategy yielded four distinct germlines: (1) dual fluorescent mutants (GFP/DsRed) expressing both Cas9 and sgRNA, (2) Nos-Cas9 line (GFP/+), (3) U6-sgRNA (DsRed/+), and (4) non-transgenic controls (fluorescence-negative) (S1 Fig). Meanwhile. the double-positive individuals (GFP/DsRed) were identified as putative mutants and selected for subsequent genotypic validation." (Lines 352-363).

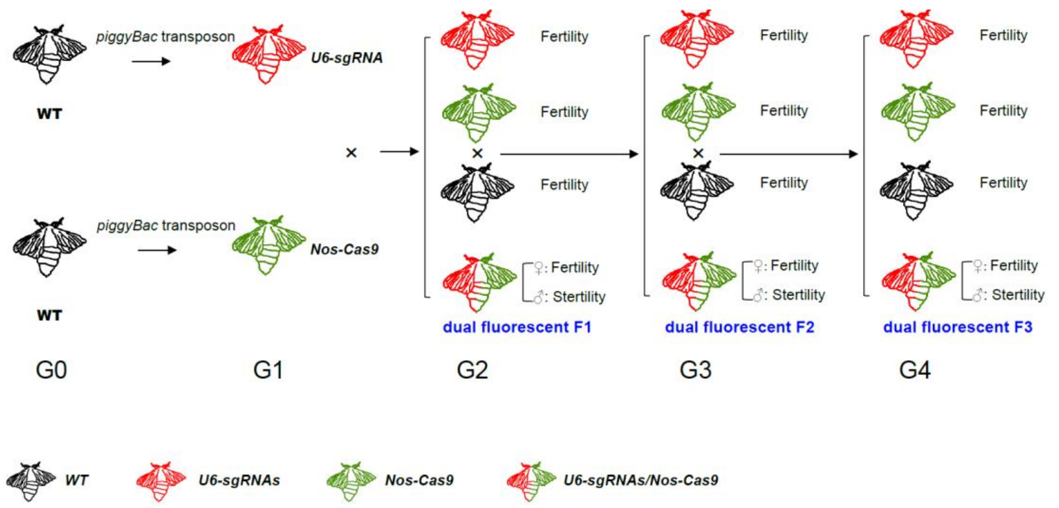

2. Following Q1: by using this approach, would the authors acquire homozygote or heterozygote TSSKL mutants? Please provide some details to support ether way.

Response: Thanks for your query. In this study, we used the binary transgenic CRISPR/Cas9 system to target the *BmTSSKL* (See S1 Fig) (Ref: 1-3). The *U6-sgRNA (TSSKL)-dsRed* and *Nos-Cas9-GFP* lines were crossed to generate the *TSSKL* mosaic mutant (F1, with dual fluorescent). Here, we use F1 (mosaic mutants) to do experiments because homozygous mutants could not be obtained due to the sterile of male mutants. In addition, the advantage of this binary transgenic system is that it allows to readily obtain mosaic mutants for experiments and observe phenotypic traits across multiple generations (F1, F2, F3, etc.). When crossing the parental lines Nos-Cas9 and U6-sgRNA (*TSSKL*), the offspring consist of four genotypes: Nos-Cas9/+ (GFP fluorescence only), U6-sgRNAs/+ (red fluorescence only), Nos-Cas9/U6-sgRNA (dual fluorescence, generating the mutant), +/+ (no fluorescence).

These four lines can freely interbreed while consistently producing the same four genotypes in subsequent generations. Notably, the male mutants in each generation remain sterile (as figure below). Overall, the mosaic mutants do not alter our key findings in our study: *TSSKL* causes defective sperm mitochondrial development, metabolic dysfunction, and male sterility.

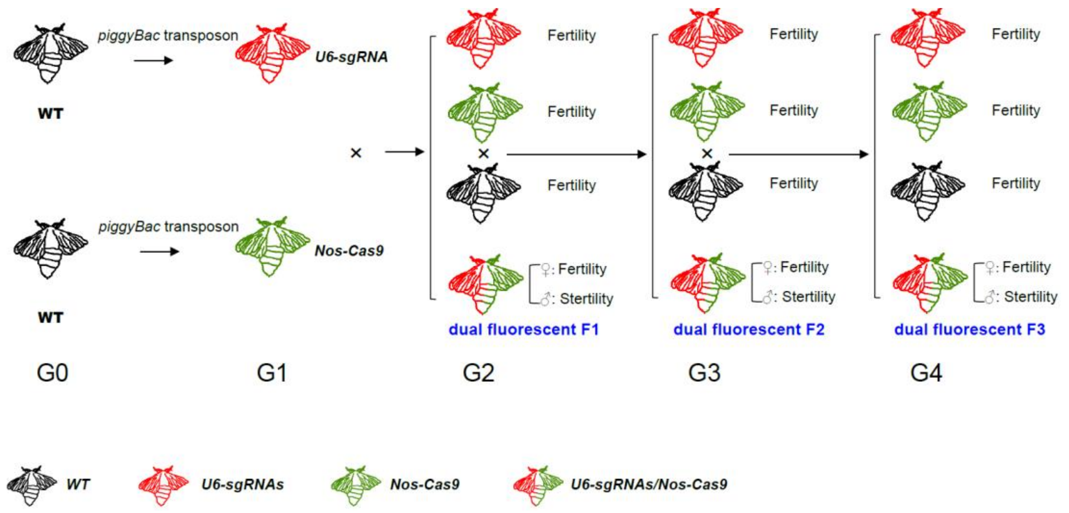

References:

1. **Functional polymorphism of CYCLE underlies the diapause variation in moths. Science. 2025, 388(6750): eado2129. doi: 10.1126/science.ado2129. PMID: 40440392.**
2. **The Prmt5-Vasa module is essential for spermatogenesis in Bombyx mori. PLoS Genetics. 2023, 19(1): e1010600. doi: 10.1371/journal.pgen.1010600. PMID: 36634107.**
3. **BmPMFBP1 regulates the development of eupyrene sperm in the silkworm, Bombyx mori. PLoS Genetics. 2022, 18(3): e1010131. doi: 10.1371/journal.pgen.1010131. PMID: 35312700.**

3. Line 168: Please include a description distinguishing undeveloped or unhatched eggs from those that developed/fertilized properly to better support the conclusion that infertility is caused by TSSKL knockout. The numbers of eggs won't lead the conclusion of infertility caused by *TSSKL* KO.

Response: We appreciate your constructive comments. Our results demonstrated that both female and male *TSSKL* mutants exhibited normal mating behavior (Fig 2E-2H). Typically, a female silkworm moth lays approximately 350 ± 20 eggs after mating, with a hatching rate of ~95%. In our egg-laying assays, both mutant and wild-type moths successfully mated. If the *TSSKL* mutation had no effect on fertility, the expected hatching rate should have remained at 95%. However, experimental groups containing male *TSSKL* mutants, the egg hatching rate was 0% (Fig 2B), indicating that the *TSSKL* mutation cause male-specific sterility. Although we did not directly examine the fertilization status of these unhatched eggs, two potential explanations emerge: i) Insufficient sperm transfer to the spermatheca, leading to fertilization failure (Fig 3B), or ii) Sperm defects rendering them incapable of fertilization (Fig 3D and 3E). Notably, while we did not directly examine the fertilization status of these eggs, this limitation does not affect our primary conclusion that *TSSKL* disruption leads to abnormal mitochondrial development and energy metabolism defects in sperm, ultimately leading to male sterility (Lines 145-148, 181-184 and 192-201). Indeed, this is an important point. It might be a good topic for our future research.

4. Please provide numbers of biological replicates for all quantitative bar graph: e.g., Fig. 2B, 3C, Fig. 4. etc

Response: Thank you for your valuable suggestion. We have already added the relevant information in the figure legends (Lines 542, 559, 577, 584, 592 and 598).

5. Line 269: "…….were significantly difference in *BmTSSKL* mutant than those of WT" change to "……were significantly different in *BmTSSKL* mutants than those of WT."

Response: Thank you. We revised the sentence as suggest (Lines 283-284).

6. Please revise conclusions and discussion to precisely interpret your study:

1) Is Nos-CAS9 driven a Nanos/Nos promoter? If so, TSSKL deletion would be expected to occur specifically in germ cells, not in somatic cells. Thus, authors cannot draw such a conclusion that "*TSSKL* knockout induces complete male sterility while preserving normal somatic development and mating behavior, demonstrating its specific requirement in post-meiotic spermatogenesis" (Line 345), as the somatic effects were not directly assessed.

Response: We appreciate this important clarification regarding the germline specificity of the *Nos-Cas9* system. The relevant information and reference were described in the Methods section (Lines 344-346). Thank you for your advice. We revised the relevant sentence in the Discussion section (Line 274).

"*TSSKL* knockout induces complete male sterility while preserving normal mating behavior."

2) Were the germ cell progenitors affected by TSSKL KO? Without this information, the authors cannot conclude that TSSKL's specific requirement in **post-meiotic** spermatogenesis.

Response: Thank you for your kind reminding. This is an important point. To ensure the manuscript's academic rigor, we have removed the relevant descriptions in the revised manuscript (Line 274).

3) The authors only presented the altered morphology of sperm but did not provide data about sperm counts in TSSKL KO. If only the functions (but not the development) of sperm were affected, the authors need to revise the conclusion that TSSKL regulates spermatogenesis.

Response: Thank you for your suggestion. We have revised the relevant sections in the revised manuscript accordingly. And it changed "spermatogenesis" to "sperm morphogenesis" (Lines 26, 49, 59, 110, 115, 128, 171, 193, 202, 206, 237, 262 and 266).

4) While it is informative to get a global transcriptome changes resulting from TSSKL KO, it is important to recognize that TSSKL is a kinase, and its deficiency primarily affects protein modification or signaling pathways rather than directly regulating gene transcription. The observed alteration in gene transcription may therefore represent downstream or indirect effects of disrupted signaling networks or targets regulated by TSSKL. Indeed, several TSSK family members have been identified in mammals and are known to influence signal transduction of germ cells. The authors should consider these alternative mechanisms and revise their conclusion that 'Our studies reveal TSSKL's central role in orchestrating mitochondrial dynamics, evidenced by its coordinated regulation of the core fusion/fission machinery.'

Response: Thank you for your advice. We have revised and improved the Discussion section in the revised manuscript (Lines 286-318).

5) The 2nd and 3rd paragraph of discussion section needs to be re-organized. The authors begin by discussing fusion and fission first, then shift to the topic to ATP synthesis and motor proteins, and then back to fusion and fission. This sequence lacks a clear logical flow and would benefit from a more cohesive structure.

Response: We sincerely appreciate your suggestion and have accordingly restructured the manuscript to improve its logical flow and clarity (Lines 286-310).
