# Supplementary material for: TSSKL is essential for sperm mitochondrial morphogenesis and male fertility in moths
Source: PLoS Genet. 2025 Oct 24;21(10):e1011914. doi: 10.1371/journal.pgen.1011914 (PMC12551868; doi:10.1371/journal.pgen.1011914)
Supplement: Attachment — Submitted filename: review.docx [file pgen.1011914.s011.docx]

In this study, Xu et al. utilized CRISPR/Cas9-mediated mutagenesis to characterize the functional role of the TSSKL gene in the lepidopteran insects. TSSKL knockout induced complete male sterility and severe morphological defects in both eupyrene and apyrene sperm. Comparative transcriptome and functional analyses linked these phenotypes to dysregulated energy metabolism pathways. Their results provided novel insights into insect reproductive biology.

The manuscript is concise, but sufficiently clear written. Experiments seem to be properly designed and precisely conducted. Results obtained are significant and well support conclusions made. I have not found any serious shortcomings, I suggest only some minor revisions.

Minor suggestions

Lines 164-166: BmTSSKL mutant males mated with WT or BmTSSKL mutant females produced eggs normally but did not hatch, whereas WT males mated with WT or BmTSSKL mutant females laid eggs and hatched normally (Fig 2A). This sentence seems to have a grammatical error.

Lines 212-214: we analyzed the mRNA expression of sperm motility genes at different developmental stages in WT and BmTSSKL mutant individuals was compared by using qRT-PCR. The meaning of this sentence seems unclear. Please check it.

For BmTSSKL, which number is the accession ID of this gene in GenBank. Please add the number in the manuscript.

In Supporting information, the file “S1 Table” was named as Table S2, “S2 Table” named as Table S3, and “S3 Table” named as Table S1. Please check them carefully.
