# Supplementary material for: TSSKL is essential for sperm mitochondrial morphogenesis and male fertility in moths
Source: PLoS Genet. 2025 Oct 24;21(10):e1011914. doi: 10.1371/journal.pgen.1011914 (PMC12551868; doi:10.1371/journal.pgen.1011914)
Supplement: Attachment — Submitted filename: PGenetic review-7-2025.docx [file pgen.1011914.s012.docx]

This study by Xu et al., identified TSSKL as an essential regulator in sperm mitochondrial morphogenesis and male fertility of moths. Their genetic and molecular experiments are well designed, and the data are presented clearly and concisely. Altogether, this is a nice study that uses two species to demonstrate the conserved role of TSSKL in sperm functions. However, I have some comments and concerns that should be addressed.

1. Line 155: The authors state, “We confirmed that gene editing was successful by crossing Nos-Cas9 line with EGFP marker (Fig. 1G). However, it means that a Nos-Cas9/GFP line was used in combination with sgRNA/dsRed to induce TSSKL deletion or mutation. This strategy seems to serve as a method for generating or selecting TSSKL knockout/KO animals, rather than as a means of confirming successful gene editing.

2. Following Q1: by using this approach, would the authors acquire homozygote or heterozygote TSSKL mutants? Please provide some details to support ether way.

3. Line 168: Please include a description distinguishing undeveloped or unhatched eggs from those that developed/fertilized properly to better support the conclusion that infertility is caused by TSSKL knockout. The numbers of eggs won’t lead the conclusion of infertility caused by TSSKL KO.

4. Please provide numbers of biological replicates for all quantitative bar graph: e.g., Fig. 2B, 3C, Fig. 4. etc

5. Line 269: “…….were significantly **difference** in *BmTSSKL* mutant than those of WT” change to “……were significantly **different** in *BmTSSKL* **mutants** than those of WT.”

6. Please revise conclusions and discussion to precisely interpret your study:

1) Is Nos-CAS9 driven a Nanos/Nos promoter? If so, TSSKL deletion would be expected to occur specifically in germ cells, not in somatic cells. Thus, authors cannot draw such a conclusion that “*TSSKL* knockout induces complete male sterility **while preserving normal somatic development and mating behavior**, demonstrating its specific requirement in post-meiotic spermatogenesis” (Line 345), as the somatic effects were not directly assessed.

2) Were the germ cell progenitors affected by TSSKL KO? Without this information, the authors cannot conclude that TSSKL’s specific requirement in **post-meiotic** spermatogenesis.

3) The authors only presented the altered morphology of sperm but did not provide data about sperm counts in TSSKL KO. If only the functions (but not the development) of sperm were affected, the authors need to revise the conclusion that TSSKL regulates spermatogenesis.

4) While it is informative to get a global transcriptome changes resulting from TSSKL KO, it is important to recognize that TSSKL is a kinase, and its deficiency primarily affects protein modification or signaling pathways rather than directly regulating gene transcription. The observed alteration in gene transcription may therefore represent downstream or indirect effects of disrupted signaling networks or targets regulated by TSSKL. Indeed, several TSSK family members have been identified in mammals and are known to influence signal transduction of germ cells. The authors should consider these alternative mechanisms and revise their conclusion that 'Our studies reveal TSSKL’s central role in orchestrating mitochondrial dynamics, evidenced by its coordinated regulation of the core fusion/fission machinery.'"

5) The 2^nd^ and 3^rd^ paragraph of discussion section needs to be re-organized. The authors begin by discussing fusion and fission first, then shift to the topic to ATP synthesis and motor proteins, and then back to fusion and fission. This sequence lacks a clear logical flow and would benefit from a more cohesive structure.
