# Supplementary material for: TSSKL is essential for sperm mitochondrial morphogenesis and male fertility in moths
Source: PLoS Genet. 2025 Oct 24;21(10):e1011914. doi: 10.1371/journal.pgen.1011914 (PMC12551868; doi:10.1371/journal.pgen.1011914)
Supplement: Attachment — Submitted filename: Response to comments_V2.docx [file pgen.1011914.s014.docx]

**Additional Editor Comments**

While the manuscript is greatly improved and the authors have addressed many concerns raised by myself and the previous reviewers, I find that the rigor in this paper still falls short of a similar study previously published in PLOS genetics by this group. Some gaps in the manuscript that I believe still need to be addressed before publication are below.

**Response: We appreciate your positive comments on our study and further suggestions that help improve the manuscript.**

1. The authors did not specify where and how many copies of the piggyBac cassettes are inserted, and how they are kept as stocks (single-insert or multicopy-insert). The best practice is to specify where the piggyBac cassette (e.g. with Splinkerette PCR) lands and keep the sgRNA and Cas9 stock as a single-copy stock. At the very least, the authors should explain the measures taken such that the transgenic individuals used for the subsequent sgRNA and Cas9 crosses do not have genes related to fertility-, sperm motility-, or mitochondria-related pathway already disrupted because of piggyBac random insertion.

**Response: Thank you for your kind reminding. We have already added the relevant information in the Methods and Results section (S1 Fig) (Lines 135-139, 382-387, and 876-879).**

**“Inverse PCR was used to determine the genomic insertion sites of the transgene as described previously. In brief, Genomic DNA was extracted from G1 transgenic moths using the CTAB method. Subsequently, 5 μg of DNA was digested with DpnII, purified and self-circularized with T4 DNA ligase. Flanking genomic regions were amplified by nested PCR using outward-facing primers specific to the 5' transposon terminal repeat (S3 Table). The resulting PCR products were sequenced to identify the insertion sites.” (Lines 382-387).**

**“Meanwhile, the insertion of the U6-sgRNAs and Nos-Cas9 transgenes was validated by inverse-PCR performed on genomic DNA isolated from the whole bodies of fluorescent G1 moths (DsRed or GFP positive) (Fig 1G). Analysis of the resulting sequences against the silkworm genome database (http://kaikoblast.dna.affrc.go.jp) thereby localized the transgenes to distinct chromosomal loci (S1 Fig).” (Lines 135-139).**

**“S1 Fig. Genomic insertion loci of the *Nos-Cas9* (A) and *U6-sgRNAs* (B) transgenic lines. The insertion sites are marked by vertical red arrows. The flanking genes are annotated with blue arrows, and the distances from the insertion sites to these genes are labeled in base pairs.” (Lines 876-879).**

2. For experimental hygiene, U6 and Cas9 transgenic line base fertility and responsive index will need to be compared to as control, instead of WT. A few experiments comparing the fertility and responsive index between WT and those lines will suffice to answer this question.

**Response: Thanks for your suggestions. To enhance the rigor of the experiment, we have included *U6-sgRNAs* and *Nos-Cas9* lines as control groups in the revised manuscript and supplemented the relevant data (Figure 2) (Lines 154, 173-179).**

3. Line 153-154: Please elaborate how the dual fluorescent F2 and F3 are made to support “male sterility inheritance”. Maybe I misunderstood this claim or the schematic – but if F2 mutants are the result of G2 green and red (which is fertile x fertile), that is not male sterility inheritance.

**Response: Thank you for your query. We apologize for any confusion in our previous response. Below, we have provided a more detailed explanation. As follows:**

**“After free hybridization of *Nos-Cas9* and *U6-sgRNAs*, four types of offspring were produced, including dual fluorescent *BmTSSKL* mutant line, Nos-Cas9 line (GFP), U6-sgRNAs line (DsRed), and non-fluorescent line (S2 Fig). Subsequently, random hybridization among these four lines only yields offspring of these same four types. Female mutants carrying the male sterility factor could mate non-mutant adult individuals and effectively reproduced the next generation. In this next generation, male mutants could mate but were sterile, while female mutants could both mate and remain fertile. In every generation of silkworms, *BmTSSKL* mutant male sterility was observed and inherited (Fig 2C).” (Lines 157-166).**

4. Figure 3A: Please add a picture reference of virgin female’s bursa copulatrix and spermatheca.

**Response: We appreciate your suggestion. We have added the relevant data and updated figure in the revised manuscript (Fig 3) (Lines 199, 580-582).**

5. Line 185-188: Please add references for these genes.

**Response: We appreciate your suggestion and have added references in the revised manuscript (Lines 201-202).**

6. Line 189-191: The BmTSSKL mutant qPCR experiments show that the BmTSSKL gene KO affect the sperm-motility genes, not that these 4 genes affect migration. This claim may be more suitable to be extrapolated in the Discussion section.

**Response: We sincerely appreciate your valuable suggestions. We have moved this sentence to the Discussion section (Lines 327-328).**

7. Figure 5E: The authors need to define the measures taken to separate unmated eggs from mated eggs if Plutella cannot be hand-mated.

**Response: Thanks for your query. To** **evaluate reproductive capacity in *Plutella xylostella*, we established four population cages (25 × 25 × 25 cm^3^) with the following crosses: 1) WT females × WT males, 2) *PxTSSKL* mutant females × WT males, 3) WT females × *PxTSSKL* mutant males, and 4) *PxTSSKL* mutant females × *PxTSSKL* mutant males. To ensure that all eggs collected were from successfully mated females, we isolated copulating pairs (as shown in S6 Fig) immediately upon observation into individual clear tubes. Egg development was subsequently assessed for these confirmed pairs.**

8. The authors show they can edit the female germline with no phenotype, so it puzzles me why they just didn’t recover a mutant from females to study. Please elaborate on this.

**Response: In our study, crossing the two transgenic lines, *Nos-Cas9* and *U6-sgRNAs*, yields four distinct progeny types: dual fluorescent *TSSKL* mutants, individuals expressing only Cas9 transgenic lines, individuals expressing only sgRNAs transgenic lines, and non-fluorescent individuals. Consequently, we can perform an initial screening for mutants based on dual fluorescence, followed by genotypic identification to rapidly prepare for subsequent large-scale experiments. The Cas9-expressing and sgRNA-expressing transgenic individuals from this generation can be crossed again to produce dual fluorescent mutants for continued experimentation. Undoubtedly, we sincerely thank you for your excellent suggestion, which has offered a highly insightful direction for our future investigations into sex-specific sterility. We really acknowledge that this particular point was overlooked in this study.**

9. Line 237: typo “sperm sperm”.

**Response: Thank you. We apologize for the mistake and have corrected it in the revised manuscript (Line 253).**
